# Surgery for a gastric Dieulafoy’s lesion reveals an occult bleeding jejunal diverticulum. A case report

**DOI:** 10.1186/1471-2482-12-S1-S29

**Published:** 2012-11-15

**Authors:** G Orlando, IM Luppino, R Gervasi, MA Lerose, B Amato, R Spagnuolo, R Marasco, P Doldo, A Puzziello

**Affiliations:** 1 Gastroenterology and Endoscopy Unit, T. Campanella Oncological Foundation, Catanzaro, Italy; 2 General Surgery Unit, Dept of General Surgery, Geriatric and Endoscopy, University Federico II, Naples, Italy; 3 Endocrine Surgery Unit, Department of Surgical and Medical Sciences, University Magna Graecia, Catanzaro, Italy

## Abstract

**Background:**

Jejunal diverticulosis is an uncommon disease and usually asymptomatic. It can be complicated not only by diverticulitis, but by hemorrhage, perforation, intussusception, volvulus, malabsorption and even small bowel obstruction due to enteroliths formed and expelled from these diverticula.

**Methods:**

We describe a case of an occult bleeding jejunal diverticulum, casually discovered in a patient that was taken to surgery for a Dieulafoy’s lesion after unsuccessful endoscopic treatment. We performed a gastric resection together with an ileocecal resection.

Macroscopic and microscopic examinations confirmed the gastric Dieulafoy’s lesion and demonstrated the presence of another source of occult bleeding in asymptomatic jejunal diverticulum.

**Discussion:**

The current case emphasizes that some gastrointestinal bleeding lesions, although rare, can be multiple and result in potentially life-threatening bleeding. The clinician must be mindful to the possibility of multisite lesions and to the correlation between results of the investigations and clinical condition of the bleeding patient.

## Background

Jejunal diverticulosis (JD) is an uncommon disease and usually asymptomatic [1] with an incidence of 0.06–1.3% although the prevalence seems to increase with age. The highest incidence of JD occurs during the sixth and seventh decades of life, and the disease is thought to be more common in males. It is frequently seen in the duodenum while jejunal and ileal locations are very rare. More commonly JD is diagnosed as an incidental finding of computed tomography imaging, small bowel barium radiographic series, or during surgery [2,3].

This report presents a case of small bowel bleeding jejunal diverticulum with enteroliths formed inside casually discovered intraoperatively as another cause of bleeding in a patient with a Dieulafoy’s lesion.

## Case report

A 75-years-old-man was admitted for an undiagnosed gastric bleeding lesion treated with endoscopic clips. He suffered of anemia and asthenia without nausea, abdominal pain or weight loss. Laboratory studies revealed mild anemia with hemoglobin level of 10 g/dL. An upper gastrointestinal endoscopy was performed showing a 2 cm bleeding ulceration at the corpo-antral junction in the greater curve. Endoscopic ultrasound (EUS) confirmed the presence of a single long vessel of 1 mm diameter perforating the muscular layer of the gastric wall and coming to lie in the submucosa. The arterial nature of the vessel was confirmed by pulse Doppler [4,5]. It was diagnosed a Dieulafoy’s lesion (DL) according to the following diagnostic criteria: 1. active arterial spurting or micropulsatile bleeding from a minute mucosal defect less than 3 mm or normal mucosa; 2. visualization of a protruding vessel with or without bleeding within a small mucosal defect or normal mucosa; 3. appearance of a fresh, adherent clot to a minute mucosal defect or normal mucosa [6]. The first choice was endoscopic treatment. Injection of epinephrine (1:10) and the use of endoscopic clips led to complete cessation of bleeding [7]. No complications developed after the procedure and there was no recurrence of bleeding at the 90-day follow-up. After six months the patient was readmitted because of a worsening of general condition with acute upper GI bleeding. An endoscopic treatment was performed placing three clips. At the follow-up the failure of procedure needed a gastric wedge resection.

**Figure 1:**
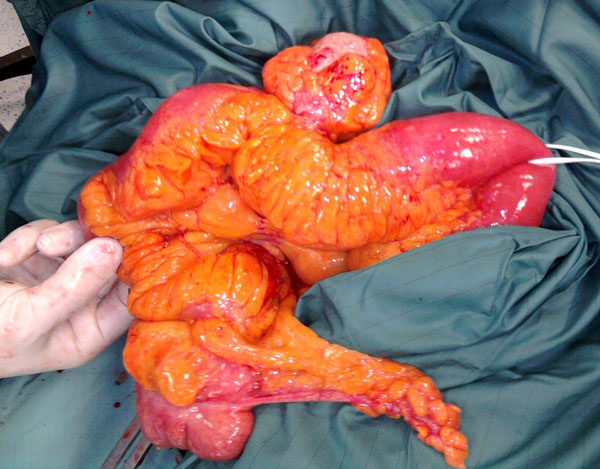
Intraoperative jejunal diverticulum

**Figure 2:**
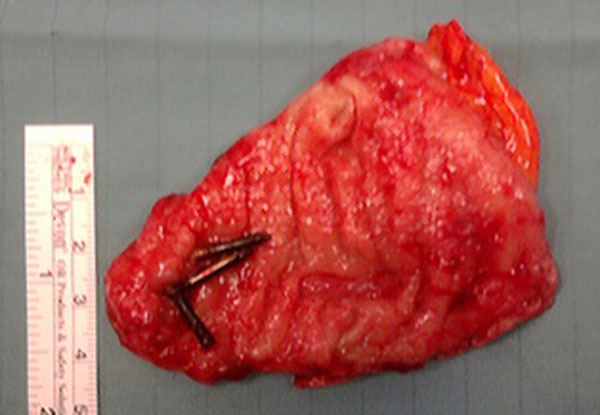
Opened wedge resection showing endoscopic clips

The patient suffered of a severe stage of COPD and laparoscopic procedure was contraindicated so the laparotomy was performed. Intraoperative abdominal exploration incidentally revealed a jejunal diverticulum close to caecum [Fig.1]. An ileocecal resection was performed with a double-layer side-to-side single stitches anastomosis (Vicryl*Plus 3/0SHplus). Afterwards, a gastric wedge resection was performed by a linear stapler (Covidien DST Series™ GIA™ 60-3.5) [Fig.2].

The discharge was in the 5^th^ day post op.

Macroscopic examination showed a segment of intestine approximately 30 cm in length with JD on the antimesenteric side. Three enteroliths were founded formed inside with a smooth dark outer surface, the largest one of 3 cm in major diameter without protruding into the intestinal lumen. Histological sections of bowel showed wall thinning of the jejunal mucosal with bleeding areas and intense neutrophilic exudate that covered all the coats of the intestinal wall. The gastric gross examination confirmed a Dieulafoy’s lesion.

One year after surgery the patient has a hemoglobin level of 14,5 g/dL.

## Discussion

This case report shows in the same patient two rare causes of GI bleeding and permits a discussion about their suitable treatment.

### Dieulafoy’s lesion

A Dieulafoy's lesion is an aberrantly dilated and tortuous submucosal arteriole, often identified after it erodes through the mucosa of the gastrointestinal tract and begins to bleed. There is no consensus on the treatment of DL. The evolution of endoscopic methods of haemostasis has markedly reduced the need for surgery achieving more than 90% of resolution [8,9]**.** These procedures can be classified into three groups: electrocoagulation, regional injection and mechanical treatments [10,11]. The initial haemostasis rate of injection therapy may be close to 95%. On the other hand, the re-bleeding after injection monotherapy is reported to be up to 55%.

More studies have shown that mechanical endoscopic methods, such as the clip placement (EHP) and the band ligation (EBL), are more effective treatments than other endoscopic methods. They could be considered the first option in the management of DL. Ahn reports in a retrospective single center study both EHP and EBL are suitable for the treatment of bleeding DL. EBL can be used as an initial haemostatic method for bleeding DL because of a favorable clinical outcome comparable to that with EHP and a shorter procedure time [12].

The risk of re-bleeding has a range of 9-40% and is higher in endoscopic monotherapy compared with combined methods. Endoscopic methods are the preferred treatment if re-bleeding occurs. Angiography, more suitable for lower GI tract lesions, can be used to stop the bleeding by selective embolisation of the feeding vessel, but up to date there are conflicting data [10]. Wide-wedge resection is today the last surgical resort overtaken by advances in endoscopic procedures. Actually, surgery has a role in the 5% of uncontrolled or unidentified bleeding and in the failure of endoscopic interventions [6,13,14].

### Jejunal diverticular disease

Jejunal diverticular disease is a rarely found pathology (±5% of post-mortem examinations). Despite being a rare pathology, there is a greater prevalence of JD disease in the older age population but it is often asymptomatic. Diverticular disease of the small intestine is noted in 60%-70% duodenal, 20%-25% jejunal, and 5%-10% ileal [2]***.*** JD is diagnosed as an incidental finding of computed tomography imaging, small bowel barium radiographic series or during surgery [3,1]. Some patients have a history of chronic symptoms such as vague abdominal discomfort, fullness, pain or recurrent central and upper abdominal cramps because of pseudo-obstruction. Anemia due to iron deficiency and megaloblastic anemia have often been reported and commonly attributed to malabsorption, steatorrhea, and B12 vitamin deficiency [3]. Patients with JD can present with emergent complications such as massive gastrointestinal bleeding, intestinal obstruction or perforation [15]. Recently, the total laparoscopic approach has been used as a valid surgical strategy, even in cases of complicated diverticulitis [16].

In this case the incidental intraoperative discovery of JD imposed the choice of an ileocecal resection as prevention of complications in a patient with an high surgical risk.

## Conclusion

In conclusion, these conditions, although rare, can be multiple and result in potentially life-threatening bleeding. The clinician must be mindful to the possibility of multisite lesions and to the correlation between results of the investigations and clinical condition of the bleeding patient. In this case the incidental discovery of an occult bleeding lesion and a targeted surgical approach made us to ensure a suitable therapy of GI bleeding. The follow up will be done by capsule endoscopy, a safe and effective method of intestinal post operative control [17].

## Competing interests

The authors declare that they have no competing interests.

## Authors' contributions

GO: conception and design, interpetration of data, given final approval of the version to be published; IML: acquisition of data, drafting the manuscript, given final approval of the version to be published; RG: acquisition of data, drafting the manuscript, given final approval of the version to be published; MAL: acquisition of data, drafting the manuscript, given final approval of the version to be published; BA: acquisition of data, drafting the manuscript, given final approval of the version to be published; RS: acquisition of data, interpretation of data, given final approval of the version to be published; RM: acquisition of data, interpretation of data, given final approval of the version to be published; PD: acquisition of data, interpretation of data, given final approval of the version to be published; AP: conception and design, critical revision, given final approval of the version to be published.
